# Casp8p41 and HIV

**DOI:** 10.18632/oncotarget.5238

**Published:** 2015-08-22

**Authors:** Nathan W. Cummins, Andrew D. Badley

**Affiliations:** Division of Infectious Diseases, Mayo Clinic Rochester, Rochester, MN, USA

**Keywords:** HIV, procaspase8, HIV protease, Casp8p41, apoptosis

With 35 million persons estimated to be living with HIV worldwide, and 2 million infections occurring each year, the HIV pandemic remains a significant public health problem. Without an effective vaccine or a cure, control of HIV relies on reliable identification of infection and institution of combination antiretroviral therapy, in order to reduce the likelihood that an infected person will transmit the disease. Acute HIV infection is characterized by a window period of negative serologic testing, and high transmissibility. After diagnosis, reliable biomarkers of risk of disease progression are lacking. Antiretroviral therapy penetrance is incomplete even in resource replete settings, and does not fully normalize life expectancy. Furthermore, viral resistance develops frequently due to the high mutation rate of the virus and failure to comply with sometimes complex medication regimens with adverse medication side effects. Therefore, there is urgent need for new targets for the diagnosis, prognosis and treatment of HIV.

Over the past fifteen years, we have described a novel pathway by which HIV kills the CD4 T cells it infects. HIV protease, which is active in the cytosol of infected cells replicating virus, cleaves the cellular apoptosis regulator procaspase8 between amino acids 355 and 356 to generate a novel cleavage fragment - Casp8p41 [1,2]. Casp8p41 translates to the mitochondria, where it initiates loss of mitochondrial outer membrane potential, release of cytochrome C and activation of caspase9, leading ultimately to apoptosis [3]. Since Casp8p41 is only generated by HIV protease cleavage, it is present only in HIV infected cells. These qualities make it ideal for potential diagnostic and prognostic uses. In fact, using a monoclonal antibody specific to the Casp8p41 C-terminal neoepitope, we have demonstrated expression of Casp8p41 in lymph nodes and circulating memory CD4 T cells in HIV infected, but not uninfected, persons [2]. In chronically infected viremic patients, Casp8p41 expression in memory CD4 T cells is inversely correlated with CD4 T cell count [4]. In these patients, decreases in Casp8p41 expression after initiation of ART better predict subsequent CD4 T cell count rise than changes in HIV RNA viral load [4]. In chronically infected patients on suppressive ART, persistent Casp8p41 expression is associated with CD4 T cell losses over time despite continued clinical virologic suppression [5]. Finally, some chronically HIV infected patients with resistant virus do not drop CD4 T cell counts as expected; we have shown that these patients harbor virus with impaired ability to cleave procaspase8 and consequently have reduced Casp8p41 production [6] yet preserved ability to cleave Gag:Pol, and thus still replicate virus. It will be of great interest to determine if measuring Casp8p41 expression levels may accurately differentiate incident versus prevalent HIV infection, since acute HIV is characterized by more infected cell death, whereas chronic infection is characterized by more uninfected bystander cell death. In addition, Casp8p41 detection may be useful in the immediate post-partum period to diagnose maternalto-child HIV transmission, as the serologic window period of current tests after perinatal transmission may delay early initiation of therapy to the newborn.

Using *in vitro* binding assays and *in silico* modeling, we have recently determined that Casp8p41 directly binds the proapoptotic Bak by a BH3-like peptide domain unmasked by HIV protease cleavage, resulting in direct activation of Bak and subsequent pore formation in the mitochondrial membrane [7]. This molecular interaction may serve as a potential therapeutic target that would be specific to HIV infected cells. Inhibiting the interaction of Casp8p41 and Bak may prevent death of HIV infected cells, thereby preserving CD4 T cell number and preventing the development of AIDS. This could be useful in situations where effective ART is not available, either due to resource limitations or antiviral resistance. On the other hand, this strategy would not address the chronic inflammation associated with untreated HIV and its survival limiting metabolic complications. In the converse situation, enhancing Casp8p41 interactions with Bak may increase death of HIV infected cells, and ultimately lead to eradication of virally infected cells and HIV cure. It is of great interest to determine ways to enhance this potentially curative effect.

Because Casp8p41 expression is specific to HIV infected cells, it may have valuable diagnostic properties that could improve current tests that are limited in certain window periods of infection. In addition, since our published data suggest that Casp8p41 is an important mediator of HIV infected cell death, it holds important prognostic information in treated and untreated disease. Finally, because it is a host-cell derived protein with a unique mechanism of action, Casp8p41 is likely a highly drugable target which would not be susceptible to the development of viral resistance.
